# Biomechanical analysis of force distribution in one-handed and two-handed child chest compression- a randomized crossover observational study

**DOI:** 10.1186/s12873-022-00566-z

**Published:** 2022-01-22

**Authors:** Jui-Yi Tsou, Chia-Lung Kao, Yi-Fang Tu, Ming-Yuan Hong, Fong-Chin Su, Chih-Hsien Chi

**Affiliations:** 1 Department of Physical Therapy, Fooyin University, Kaohsiung, Taiwan; 2 Department of Emergency Medicine, National Cheng Kung University Hospital, College of Medicine, National Cheng Kung University, Tainan, Taiwan, No. 138 Sheng-Li Road, Tainan City, 70403 Taiwan; 3 Department of Pediatrics, National Cheng Kung University Hospital, College of Medicine, National Cheng Kung University, Tainan, Taiwan; 4 Department of Biomedical Engineering, National Cheng Kung University, Tainan, Taiwan

**Keywords:** Biomechanics, Cardiopulmonary resuscitation, Chest compression, Child

## Abstract

**Background:**

**E**ven force distribution would generate efficient external chest compression (ECC). Little research has been done to compare force distribution between one-hand (OH) and two-handed (TH) during child ECC. Therefore, this study was to investigate force distribution, rescuer perceived fatigue and discomfort/pain when applying OH and TH ECC in children.

**Methods:**

Crossover manikin study. Thirty-five emergency department registered nurses performed lone rescuer ECC using TH and OH techniques, each for 2 min at a rate of at least 100 compressions/min. A Resusci Junior Basic manikin equipped with a MatScan pressure measurement system was used to collect data. The perceived exertion scale (modified Borg scale) and numerical rating scale (NRS) was applied to evaluate the fatigue and physical pain of delivering chest compressions.

**Results:**

The maximum compression force (kg) delivered was 56.58 ± 13.67 for TH and 45.12 ± 7.90 for OH ECC (*p* <  0.001). The maximum-minimum force difference force delivered by TH and OH ECC was 52.24 ± 13.43 and 41.36 ± 7.57, respectively (*p* <  0.001). The mean caudal force delivered by TH and OH ECC was 29.45 ± 16.70 and 34.03 ± 12.01, respectively (*p* = 0.198). The mean cranial force delivered by TH and OH ECC was 27.13 ± 11.30 and 11.09 ± 9.72, respectively (*p* <  0.001). The caudal–cranial pressure difference delivered by TH and OH ECC was 19.14 ± 15.96 and 26.94 ± 14.48, respectively (*p* = 0.016). The perceived exertion and NRS for OH ECC was higher than that of the TH method (*p* < 0.001, *p* = 0.004, respectively).

**Conclusions:**

The TH method produced greater compression force, had more efficient compression, and delivered a more even force distribution, and produced less fatigue and physical pain in the rescuer than the OH method.

**Trial registration:**

The Cheng Kung University Institutional Review Board A-ER-103-387. http://nckuhirb.med.ncku.edu.tw/sitemap.php

## Background

Child basic life support (BLS) guidelines are applicable to children from the age of approximately 1 year until puberty. When performing cardiopulmonary resuscitation (CPR) on most children, either one or two hands can be used to compress the chest [1–5].

In pediatric manikin studies, two-handed (TH) external chest compression (ECC) will produce a greater compression depth, [6] larger intrathoracic pressure, [7] and less rescuer fatigue, [8] and insufficient recoil than one-handed (OH) ECC [6]. Peska et al. suggested TH has a better balance control [6]. A better balance control could be related to more even force distribution during ECC. Although even force distribution would produce effective resultant force, labor-saving, and to be easier and comfort for ECC, little research has been done to compare force distribution between OH and TH ECC.

ECC is a cyclic movement of compression and decompression. Different forces distribution across the rescuers’ hands were produced according to the different methods of chest compression [7]. This differences could further affect the performance of CPR. Therefore, the purpose of this study was to investigate force distribution, rescuer perceived fatigue and discomfort/pain when applying OH and TH ECC in children.

## Methods

### Study design

A randomized crossover study.

### Participants

Thirty-five emergency department CPR-certified and registered nurses voluntarily participated in this study. Participants was professionals in first aid related work and had CPR certification. No participant had any muscular skeletal injury, sprain, or pain. Participants were not allowed to eat within 30 min of the tests. Consuming alcohol, tea, or coffee was prohibited on the days of the test. This simulation study was approved by the Cheng Kung University Institutional Review Board. All subjects provided written informed consent.

### Equipment

A Resusci Junior Basic and SkillGuide manikin (Laerdal, Stavanger, Norway) was equipped with a MatScan pressure measurement system (Tekscan Inc., South Boston, USA), which was applied to a Junior Basic manikin and used to record the delivered force at a sampling frequency of 30 Hz. The MatScan consists of 2288 pressure sensors aligned in 44 rows and 52 columns, with a spatial resolution of 1.4 sensors/cm^2^. The sensors are paper thin, lightweight, and flexible. The system has displayed high accuracy and moderate to good reliability [8, 9].

### The perceived exertion scale and numerical rating scale

Two subjective scales, the perceived exertion scale (modified Borg scale) and the numerical rating scale (NRS), were applied for rating the perceived fatigability of chest compression delivery and physical pain or discomfort, respectively.

The ratings of perceived exertion (RPE) were given using a modified Borg scale which had been validated to estimate the instantaneous fatigue status of the muscle in tasks [10]. It with scores ranging from 0 to 10, where, for example, 0 represents no fatigue at all, 3 represents moderate fatigued, 5 represents very fatigued, 7 represents nearly exhausted and 10 represents absolutely exhausted [10].

The NRS is an 11-point scale comprising a number from 0 through 10; 0 indicates “no pain”, and 10 indicates the “worst imaginable pain”. Patients were instructed to choose a single number from the scale that best indicates their level of pain [11].

In studies, the NRS and Borg scale have exhibited good validity and reliability [11–14].

### Procedure

The participants practiced on manikins before they began the tests to familiar with CPR skill.

Each participant performed child BLS using both TH and OH ECC in random order using a computer-generated random table [15]. A lone rescuer administered compressions and ventilations at a ratio of 30:2, delivering compressions at a rate of at least 100 compressions/min. An audio prompt was used to keep participants on pace to deliver an adequate rate of compression, namely 110/min, and a visual prompt was used to keep participants on target to deliver a suitable compression depth [2]. To ensure the quality of chest compressions and mitigate the stress of coordinating ventilation and compression efforts, specific breaks were provided for performing ventilation [16]. A 4-s pause was added to replace ventilation between each set of 30 chest compressions to simulate the actual practice of CPR. Participants performed each technique of ECC for 2 min, with a rest period of 30 min between sessions [16]. The delivered force was recorded during 2 min ECC. Physiological parameters, including heart rate and blood pressure, of each participant were measured before and after each ECC session. At the end of every test of ECC delivery, the participants were asked to rate the RPE and NRS immediately.

### Data analysis

During the 2-min CPR sessions, compression pressure was recorded. The maximum pressure, maximum and minimum force over the entire compression area, the cranial area, and the caudal area were calculated. The nipple line of the manikin separated the entire compression area into the cranial and caudal areas [7].

A sample size calculation was performed using G*Power [17] based on the results of a pilot study comprising eight subjects. We used the mean and SD of difference (3.25 ± 7.11 kg) of the primary outcome variable corresponding to the caudal-cranial force difference. A total sample size of 31 is required to achieve 80% power and the calculated effect size of 0.46 at an alpha level of 0.05. Considering a potential attrition rate of 10%, we concluded that 35 participants were necessary.

We used descriptive statistics to present outcome variables. The difference between the TH and OH techniques were analyzed using paired t-tests for continuous variables, or Wilcoxon signed rank test for continuous variables without normal distribution and ordinal variables. The significance level was set at *p* < 0.05. The data were analyzed using SPSS version 17 (SPSS Corp., Armonk, NY, USA).

## Results

Thirty-five CPR-certified RNs voluntarily participated in the study. Among them, 28 were women. The mean age of the rescuers was 27.8 ± 4.0 years, the mean height was 163.0 ± 7.4 cm, the mean weight was 61.4 ± 14.0 kg, and the mean CPR related work experience was 3.8 ± 3.5 years.

### Pressure distribution

**Fig. 1:**
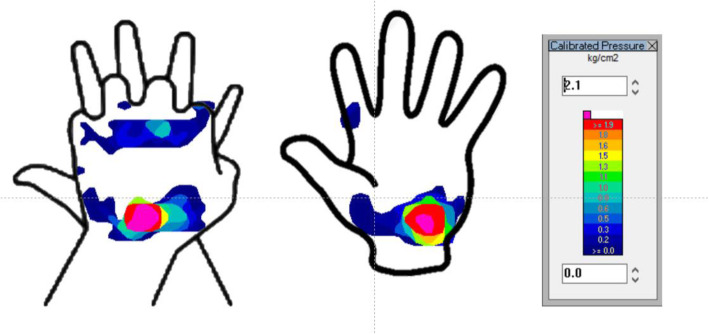
The hand position placement relates to pressure mapping of the palm in a sample of one person. Color gradient shows different pressure levels

Figure 1 illustrates the hand position placement and pressure mapping of the palm in a sample of one person. Table 1 presents a summary of palm caudal pressure, cranial pressure, and the caudal–cranial pressure gradient exerted in the two methods. Significant differences between the two compression methods were evident in the pressure delivered to the cranial and caudal areas. The mean caudal pressure was 1.78 ± 0.64 kg/cm^2^ in TH ECC and 2.13 ± 0.56 kg/cm^2^ in OH ECC, with a mean difference (95% confidence interval [CI] of difference) of − 0.35 (− 0.59 to − 0.11), *p* = 0.006. The mean cranial pressure was 1.56 ± 0.65 kg/cm^2^ in TH ECC and 1.23 ± 0.77 kg/cm^2^ in OH ECC, with a mean difference (95% CI of difference) of 0.33 (0.33–0.64), *p* = 0.031. The paired comparison revealed that while the maximum pressure and caudal–cranial pressure differences were not significant, *p* = 0.227 and *p* = 0.083, respectively.

**Table 1:** The palm’s maximum pressure, caudal pressure, cranial pressure and caudal-cranial pressure gradient in Two-handed and one-handed child ECC (kg/cm^2^)

Pressure	Two-handed	One-handed	Mean difference (95% CI of difference)	*P* value
	Mean ± SD	Mean ± SD		
Max. Pressure (kg/cm^2^)	2.04 ± 0.50	2.18 ± 0.56	−0.14(− 0.37 ~ 0.09)	0.227
Caudal Pressure (kg/cm^2^)	1.78 ± 0.64	2.13 ± 0.56	− 0.35(− 0.59 ~ − 0.11)	0.006*
Cranial Pressure (kg/cm^2^)	1.56 ± 0.65	1.23 ± 0.77	0.33(0.33 ~ 0.64)	0.031*
C-C Pressure Difference (kg/cm^2^)	0.65 ± 0.58	0.96 ± 0.75	−0.30(− 0.64 ~ 0.04)	0.083

* *p* < 0.05, significant difference

*ECC* External chest compression, *C-C* Caudal-cranial

### Force distribution

Table 2 presents the compression force delivered by the TH and OH methods. A paired comparison of maximum force indicates that the compression force delivered by the TH method was significantly higher than that delivered by the OH method (*p* < 0.001) and minimum (residual) force was not significant difference (*p* = 0.970). The Maximum-Minimum force difference presented the efficient compression force was the TH method was significantly higher than the OH method (*p* < 0.001). The caudal side force delivered in TH ECC was similar to that delivered in OH ECC (*p* = 0.198). The cranial side force delivered in TH ECC was greater than that in OH ECC (*p* < 0.001). TH ECC delivered a smaller caudal–cranial force than did OH ECC (*p* = 0.016). The results indicated that the compression method affects the force delivered and force distribution. The TH method produced more force, whereas the OH method produced a greater caudal–cranial force difference. This causes force to be distributed more toward the manikin’s head.

**Table 2:** Maximum force and difference in two-handed and one-handed child ECC (kg)

Force	Two-handed	One-handed	Mean difference (95% CI of difference)	*P* value
	Mean ± SD	Mean ± SD		
Max. Force (Kg)	56.58 ± 13.67	45.12 ± 7.90	11.45(6.74 ~ 16.17)	< 0.001*
Caudal Force (Kg)	29.45 ± 16.70	34.03 ± 2.01	−4.59(− 11 ~ 2.52)	0.198
Cranial Force (Kg)	27.13 ± 11.30	11.09 ± 9.72	16.04(10.90 ~ 21.17)	< 0.001*
C-C Force Difference (Kg)	19.14 ± 15.96	26.94 ± 14.48	−7.80(−14.03 ~ − 1.57)	0.016*
Min.(residual) Force (Kg)	4.34 ± 3.36	3.76 ± 3.65	0.58(− 0.64 ~ 1.800	0.970
Max.-Min. Force Difference	52.24 ± 13.43	41.36 ± 7.57	10.87(5.73 ~ 16.01)	< 0.001*

* *p* < 0.05, significant difference

*ECC* External chest compression, *C-C* Caudal-cranial

### Rescuer physiological parameters before and after performing ECC

A comparison of physiological variables (Table 3) before and after administering ECC indicates that heart rate (HR) and systolic blood pressure (SBP) increased significantly in response to administering both ECC techniques. Diastolic blood pressure (DBP) and mean arterial pressure (MAP), however, did not change significantly within-group for either technique. Between-group differences in the changes in SBP, DBP, MAP, and HR were not significant (*p* = 0.385, *p* = 0.102, *p* = 0.576, and *p* = 0.653, respectively).

**Table 3:** Rescuers’ physiologic parameters before and after performing ECC

	Before ECC Mean ± SD	After ECC Mean ± SD	Mean difference (95% CI of the difference)	*p*-value
*Two-handed ECC*				
Systolic blood pressure	117.64 ± 14.95	126.50 ± 13.81	− 8.86 (− 12.56 ~ − 5.16)	< 0.001*
Diastolic blood pressure	77.55 ± 76.07	76.07 ± 8.53	1.47 (− 0.90 ~ 3.85)	0.218
Mean arterial pressure	90.90 ± 10.77	92.88 ± 9.60	− 1.97 (− 4.35 ~ 4.13)	0.103
Heart rate	76.66 ± 0.02	86.11 ± 12.90	−9.46 (− 6.38 ~ − 6.22)	< 0.001*
*One-handed ECC*				
Systolic blood pressure	119.40 ± 14.49	125.58 ± 15.83	− 6.17 (− 10.54 ~ − 1.81)	0.007*
Diastolic blood pressure	76.24 ± 8.63	77.93 ± 9.82	− 1.68 (− 4.40 ~ 1.02)	0.217
Mean arterial pressure	98.03 ± 4.97	93.81 ± 10.76	4.22 (− 9.88 ~ 1.83)	0.550
Heart rate	77.58 ± 9.43	87.91 ± 14.46	− 10.33 (− 13.58 ~ − 7.08)	< 0.001*

*ECC* external chest compression

* *p* < 0.05, significant difference

### Subjective RPE, level of fatigue, and discomfort

Table 4 indicates that perceived exertion of administering TH ECC was lower than that of administering OH ECC. The median perceived exertion score for both TH and OH was 4.5 and 5. Two min of child ECC was considered a “heavy” exercise. The overall discomfort of performing TH and OH ECC, measured using the NRS, was 4 and 5, respectively (*p* = 0.008). Participants experienced pain or discomfort during the test, which was most frequently localized in the wrist. The incidence of wrist pain when performing TH and OH ECC was 58 and 80%, respectively. The intensity of wrist pain in TH and OH ECC, measured using the NRS, was 3 and 5, respectively (*p* = 0.004). The compression discomfort when performing TH and OH ECC, measured using the NRS, was 4 and 5, respectively (*p* = 0.003). No significant difference in decompression discomfort, measured using the NRS, was identified between the two groups (*p* = 0.680). In OH ECC, the hand used is prone to tiring, and thus, a rescuer is more likely to have hand and body discomfort.

**Table 4:** Subjective Rating of Perceived Exertion (RPE) and discomfort data between two-handed and one-handed techniques

	Two-handed	One-handed	*p*-value
	Median (Range)	Median (Range)	
RPE (Modified Borg Scale)	4.5 (0 ~ 8)	5 (0 ~ 10)	0.003*
Overall discomfort/pain (NRS)	4 (2 ~ 7)	5 (2 ~ 10)	0.008*
Wrist discomfort/pain (NRS)	3 (0 ~ 8)	5 (0 ~ 10)	0.004*
Compression discomfort/pain (NRS)	4 (0 ~ 7)	5 (2 ~ 10)	0.003*
Decompression discomfort/pain (NRS)	0 (0 ~ 4)	2 (0 ~ 5)	0.680

* *p* < 0.05, significant difference

*ECC* External chest compression, *NRS* Numerical rating scale

## Discussion

This study assessing the difference in OH and TH ECC as related to pressure generated and performer perception. Study participants performed child BLS by using TH and OH ECC at a standard compression rate and depth with audio and visual prompts. The results indicated that the compression method affects the force delivered and force distribution. The TH method produced more force, whereas the OH method produced a greater caudal–cranial force difference. This causes the force to be distributed more toward the manikin’s head, which might increase the risk of fractures during ECC [7, 18]. From the evaluation of the two methods of ECC currently recommended under BLS guidelines, When the providers performed high quality ECCs i.e. adequate ECC in rate, depth, and recoil based on audiovisual feedback, TH compression is superior to OH in delivering force and distributing pressure evenly over the Resusci Junior Basic manikin and also induces less fatigue and physical discomfort in rescuers. The results can be used as a reference for subsequent child ECC quality improvement and training.

Previous child manikin ECC studies have determined that participants appreciated the higher chest compression performance obtained and the lessened rescuer fatigue that result from applying the TH technique [6, 19]. Biomechanical studies further supports that ECC cyclic movement produces force distribution across the heel, and different chest interface contact approaches may influence compression force transmission during ECC [18, 20].^,^ Another study indicated that TH ECC produces significantly higher mean and peak intrathoracic pressures than does OH ECC [19]. No significant difference in peak pressure was identified between the TH and OH methods in our study, but significant differences were apparent in the maximum force, maximum–minimum force difference, caudal pressure, cranial pressure, and caudal–cranial force differences between TH and OH ECC. *A more* ergonomic type of exertion can help to prevent *fatigue* and discomfort. That the even force distribution resulted in less fatigue might explain why the majority of participants preferred the TH compression technique [6, 19]. Several researchers have reported that TH ECC was considered to have incomplete chest recoil when compared to OH ECC [1, 21]. In our study, although the difference was not statistically significant, *there* was a *trend* that TH has greater residual force than OH. Nevertheless, greater efficient compression force (maximum-minimum force difference) indicated TH had larger compression force than OH. It suggests that TH has a superior efficient compression, which might be able to compensate the incomplete recoil.

The joint most commonly affected by pain among rescuers performing ECC was the wrist. This finding is consistent with previous studies [22–24]. Curran and colleagues determined that the wrist being cyclically in positions of hyperextension, ulnar deviation, and intercarpal supination during ECC may cause damage to the scapholunate ligament of a rescuer’s wrist [25]. Here, OH generated a lower maximum force but caused more wrist pain. Wrist pain may influence force of delivery of ECC or its quality; Peska et al. found that the compression rate decreases more quickly when the rescuer uses the OH technique [6]. Compared with TH ECC, we determined that OH ECC produces more uneven force and force distribution, which may also be a risk factor for wrist pain.

### Limitations

This study has limitations. This is a manikin study, which may not reflect actual patients. The amount of force needed to move a fixed mannequin’s chest seems a great deal more than for an actual child so that the increased force able to be generated in this model may be too much and cause more fatigue than an actual patient. The choice of OH vs. TH is something ECC provider must consider in the 1–8 year old. This age gap represents a huge variation in chest wall size and recoil yet the measurements are done on one mannequin whose size likely more resembles the older child and so may not reflect the age group we often do one handed ECCs on—the younger child.

The authors compared measuring forces and pressures between two different methods of chest compression. However, there were no data on the performance of CPR such as average chest compression depth and rate, average ventilation volume and hands-off time (or chest compression fraction). Although comparisons of force or pressure between two different methods of chest compression are important, we should know whether the differences of pressure or force could affect the performance of CPR or not. As a result, we cannot determine whether the differences in the forces or pressures could affect the quality of CPR. We did not measure the performance of CPR. However, to ensure that the pressure distribution is observed under comparable condition and qualities, we provided visual and auditory feedbacks during ECC. Previous studies found real-time feedbacks may serve as a useful adjunct to guide the compression depth for rescuers during ECC [7, 26]. Our results may not be generalizable to other ECC providers especially given the small number of participants, mostly female which would make it hard to assess for confounders like gender, size, height.

## Conclusions

Our biomechanical analysis indicated that TH ECC delivers a smoother force, more even pressure distribution and more efficient compression while inducing less fatigue and discomfort/pain in the rescuer than does OH child ECC. If possible, TH should be applied rather than OH for child ECC during CPR to optimize biomechanical efficiency and rescuer comfort.

## Data Availability

The datasets used and/or analyzed during the current study are available from the corresponding author on reasonable request.

## Abbreviations

BLS: Basic life support
CPR: Cardiopulmonary resuscitation
OH: One-handed
TH: Two-handed
ECC: External chest compression
NRS: Numerical rating scale
RPR: Ratings of perceived exertion
HR: Heart rate
SBP: Systolic blood pressure
DBP: Diastolic blood pressure
MAP: Mean arterial pressure
