# Correction: Biomechanical analysis of force distribution in one-handed and two-handed child chest compression- a randomized crossover observational study

**DOI:** 10.1186/s12873-022-00702-9

**Published:** 2022-09-01

**Authors:** Jui-Yi Tsou, Chia-Lung Kao, Yi-Fang Tu, Ming-Yuan Hong, Fong-Chin Su, Chih-Hsien Chi

**Affiliations:** 1 Department of Physical Therapy, Fooyin University, Kaohsiung, Taiwan; 2 Department of Emergency Medicine, National Cheng Kung University Hospital, College of Medicine, National Cheng Kung University, Tainan, Taiwan, No. 138 Sheng-Li Road, Tainan City, 70403 Taiwan; 3 Department of Pediatrics, National Cheng Kung University Hospital, College of Medicine, National Cheng Kung University, Tainan, Taiwan; 4 Department of Biomedical Engineering, National Cheng Kung University, Tainan, Taiwan

**Correction: BMC Emerg Med 22, 13 (2022)**

**https://doi.org/10.1186/s12873-022-00566-z**

The original article [1] contained an error in the affiliation of corresponding author, Chih-Hsien Chi. The correct affiliation has been re-implemented, and is as follows:

"Department of Emergency Medicine, National Cheng Kung University Hospital, College of Medicine, National Cheng Kung University, Tainan, Taiwan, No. 138 Sheng-Li Road, Tainan City, 70403, Taiwan."
